# Reduced functional capacity is associated with the proportion of impaired myocardial deformation assessed in heart failure patients by CMR

**DOI:** 10.3389/fcvm.2023.1038337

**Published:** 2023-02-09

**Authors:** Djawid Hashemi, Patrick Doeblin, Moritz Blum, Karl Jakob Weiss, Matthias Schneider, Rebecca Beyer, Burkert Pieske, Hans-Dirk Duengen, Frank Edelmann, Sebastian Kelle

**Affiliations:** ^1^Department of Internal Medicine and Cardiology, Charité – Universitätsmedizin Berlin, Berlin, Germany; ^2^Department of Internal Medicine and Cardiology, German Heart Institute Berlin, Berlin, Germany; ^3^German Centre for Cardiovascular Research, Partner Site Berlin, Berlin, Germany; ^4^Brookdale Department of Geriatrics and Palliative Medicine, Icahn School of Medicine at Mount Sinai, New York, NY, United States

**Keywords:** heart failure, cardiovascular magnetic resonance imaging, myocardial deformation, quality of life, CMR, score, strain, quantitative

## Abstract

**Aims:**

Heart failure (HF) does not only reduce the life expectancy in patients, but their life is also often limited by HF symptoms leading to a reduced quality of life (QoL) and a diminished exercise capacity. Novel parameters in cardiac imaging, including both global and regional myocardial strain imaging, promise to contribute to better patient characterization and ultimately to better patient management. However, many of these methods are not part of clinical routine yet, their associations with clinical parameters have been poorly studied. An imaging parameters that also indicate the clinical symptom burden of HF patients would make cardiac imaging more robust toward incomplete clinical information and support the clinical decision process.

**Methods and results:**

This prospective study conducted at two centers in Germany between 2017 and 2018 enrolled stable outpatient subjects with HF [*n* = 56, including HF with reduced ejection fraction (HFrEF), HF with mid-range ejection fraction (HFmrEF), and HF with preserved ejection fraction (HFpEF)] and a control cohort (*n* = 19). Parameters assessed included measures for external myocardial function, for example, cardiac index and myocardial deformation measurements by cardiovascular magnetic resonance imaging, left ventricular global longitudinal strain (GLS), the global circumferential strain (GCS), and the regional distribution of segment deformation within the LV myocardium, as well as basic phenotypical characteristics including the Minnesota Living with Heart Failure Questionnaire (MLHFQ) and the 6-minute walk test (6MWT). If less than 80% of the LV segments are preserved in their deformation capacity the functional capacity by 6MWT (6 minutes walking distance: MyoHealth ≥ 80%: 579.8 ± 177.6 m; MyoHealth 60–<80%: 401.3 ± 121.7 m; MyoHealth 40–<60%: 456.4 ± 68.9 m; MyoHealth < 40%: 397.6 ± 125.9 m, overall *p*-value: 0.03) as well as the symptom burden are significantly impaired (NYHA class: MyoHealth ≥ 80%: 0.6 ± 1.1 m; MyoHealth 60–<80%: 1.7 ± 1.2 m; MyoHealth 40–<60%: 1.8 ± 0.7 m; MyoHealth < 40%: 2.4 ± 0.5 m; overall *p*-value < 0.01). Differences were also observed in the perceived exertion assessed by on the Borg scale (MyoHealth ≥ 80%: 8.2 ± 2.3 m; MyoHealth 60–<80%: 10.4 ± 3.2 m; MyoHealth 40–<60%: 9.8 ± 2.1 m; MyoHealth < 40%: 11.0 ± 2.9 m; overall *p*-value: 0.20) as well as quality of life measures (MLHFQ; MyoHealth ≥ 80%: 7.5 ± 12.4 m; MyoHealth 60–<80%: 23.4 ± 23.4 m; MyoHealth 40–<60%: 20.5 ± 21.2 m; MyoHealth < 40%: 27.4 ± 24.4 m; overall *p*-value: 0.15)–while these differences were not significant.

**Conclusion:**

The share of LV segments with preserved myocardial contraction promises to discriminate between symptomatic and asymptomatic subjects based on the imaging findings, even when the LV ejection fraction is preserved. This finding is promising to make imaging studies more robust toward incomplete clinical information.

## 1. Introduction

Patients with heart failure (HF) are at high risk for mortality and hospitalization and have a high burden of symptoms that alter their function and health-related quality of life (QoL) (1–5). QoL and functional capacities contributing to QoL are major goals in monitoring and treating patients with HF. Although patients with HF and a low QoL may have a higher potential for improvement, they may also be in a stage of the disease that is too advanced to improve (6). Therefore it is important to measure other contributing parameters associated with QoL, disease state and prognosis to better assess the patients (7). QoL measures are often evaluated in clinical trials as patient reported outcome measures become increasingly important, but they are rarely implement in clinical routine. Hence, identifying routine measures reflecting insights into QoL as well as functional capacities will contribute to an improved assessment of HF patients.

Various dimensions including physical capacity influence QoL. Routine measures to assess physical capacities include the semi-objective 6-minute walk tests and the subjective New York Heart Association (NYHA) functional class, often used in clinical trials but rarely in daily routine (8).

Cardiovascular magnetic resonance imaging (CMR) is a comprehensive technique, increasingly accessible and providing not only a high resolution of information on functional cardiac parameters but also information of tissue characteristics. Measurements of cardiac contractility and the assessment of myocardial deformation by strain analyses are an emerging and promising tool to better characterize patients compared to traditional parameters, e.g., left ventricular ejection fraction (LVEF) (9). While LVEF as well as routine strain measurements provide a global impression of cardiac contractility, recent studies showed the relevance of both myocardial deformation *per se* and the distribution of its impairment assessed by strain measurements characterizes HF patients in more detail (10). The added value of strain compared to LVEF in HF has been highlighted in HFpEF where LVEF is preserved. The MyoHealth score has been introduced as a parameter highlighting the heterogeneity of regions with altered myocardial deformation compared to preserved regional myocardial strain values (10–12). The score is calculated by the ratio of LV segments with preserved myocardial deformation to the total number of LV segments in a 37 segment LV model (10). It has been shown that cardiac remodeling does not present itself simultaneously across all LV segments in HF (10). Various reasons lead to the regional differences of both systolic and diastolic changes including shearing stress induced diffuse fibrosis, altered local gene expression patterns or global metabolic changes of cardiomyocytes (13–16).

We hypothesize that the better characterization of cardiac deformation in HF patients provides information on QoL and functional capacity. Therefore, we aim to evaluate the association of cardiac deformation assessed by the MyoHealth score and parameters for QoL and functional capacity in this analysis.

## 2. Materials and methods

This study was a prospective study conducted at two centers in Berlin, Germany, the Charité—University Medicine Berlin and the German Heart Centre Berlin, between 2017 and 2018. Its rationale and design have been previously described (10, 17–20).

Briefly, subjects were screened for diagnosed HF and an age of at least 45 years. The initial diagnosis of HF should have been older than 30 days; the patients were required to be in a stable state with no changes in their HF medication and no HF hospitalization within the previous 7 days. HFrEF was defined as diagnosis of HF, increased N terminal pro brain natriuretic peptide (NT-proBNP) (>220 pg/mL) and LVEF < 40%, HFmrEF as the diagnosis of HF, increased NT-proBNP (>220 pg/mL) and 40% ≥ LVEF < 50% as well as HFpEF as diagnosis of HF, increased NT-proBNP (>220 pg/mL) and LVEF ≥ 50% at the time of study inclusion. We did not distinguish between the causes for HF for recruiting patients (10).

Additionally, we recruited subjects without HF or advanced cardiovascular (CV) diseases as controls.

All studies included complied with the Declaration of Helsinki, the protocols were approved by the responsible ethics committees, and all patients gave written informed consent. It was registered at the German Clinical Trials Register (DRKS, registration number: DRKS00015615). The detailed inclusion and exclusion criteria are listed on the webpage of the DRKS.

### 2.1. Cardiac magnetic resonance

As previously described, all CMR images were acquired using a 1.5 T (Achieva, Philips Healthcare, Best, The Netherlands) magnetic resonance imaging (MRI) scanner with a five-channel cardiac surface coil in a supine position. All study participants were scanned using the identical comprehensive imaging protocol. The study protocol included initial scouts to determine cardiac imaging planes. Cine images were acquired using a retrospectively gated cine-CMR in cardiac short-axis, vertical long-axis, and horizontal long-axis orientations using a steady-state free precession sequence for volumetry (10, 20). The calculation of the cardiac indices (CIs) is based on the volumetry of the ventricles. Fast strain-encoded (fast-SENC) MRI was used for strain evaluation, as it has been shown to enable quantification of longitudinal and circumferential strain in free breathing and with high reproducibility (21). Images were blinded to strain analysis, cine, and volumetric measurements, respectively. We waived reproducibility analyses based on an analysis that highlighted the robustness of fast-SENC analyses regarding intraobserver and reproducibility variabilities (22).

### 2.2. Image analysis

All images were analyzed offline using commercially available software in accordance with the recent consensus document for quantification of LV function using CMR (23). In the analysis, we included 2 chamber, 3chamber, and 4chamber cine images, and respectively, three preselected mid-ventricle slices from the LV short-axis stack. Image analysis was performed using the software Medis^®^ Suite MR (Medis medical imaging systems, Leiden, The Netherlands, version 3.1) for voluinterme measurements and the software MyoStrain (Myocardial Solutions, Inc., Morrisville, NC, USA, version 5.0) for fast-SENC strain measurements.

### 2.3. Endpoints

The study population was not only categorized by traditional HF entities, but also by the ratio of myocardial segments with preserved deformation to the total number of myocardial segments (*n* = 37), described as MyoHealth score (illustrated in S1 of Supplementary material; 10–12). Briefly, the MyoHealth score assess the 37 segments of the LV separately, whether the myocardial deformation is altered, i.e., whether the strain value of that segment is >−17%. The MyoHealth score is the proportion of LV segments with preserved and not altered myocardial deformation from the total 37 segments. The MyoHealth entities introduced include 4 groups: MyoHealth > 80%, MyoHealth 60–<80%, MyoHealth 40–<60%, and MyoHealth < 40%.

Based on the MyoHealth score distribution following parameters were assessed: QoL, 6-minute walk test (6MWT) key parameters as well as the New York Heart Association (NYHA) functional classification.

Patients were instructed to cover the maximum distance in 6 min (6-minute walk distance, 6MWD) at a self-graded walking speed, pausing to rest when needed. The test was supervised by the same study staff to minimize the variability. The 6MWD as well as the level of perceived exertion indicated as specific level on the Borg score were recorded. The functional capacities indicated by the New York Heart Association (NYHA) functional classification were also part of the baseline information collected.

In accordance with the study protocol, study participants completed a QoL questionnaire, the Minnesota Living with Heart Failure Questionnaire (MLHFQ) (24).

### 2.4. Statistical analysis

Statistical analysis was carried out with R version 3.5.1 (R Foundation for Statistical Computing, Vienna, Austria).

Normality of variables was assessed by visual assessment of normality curves and the Shapiro–Wilk test. Comparison between groups for continuous variables was performed with a one-way ANOVA for normally distributed data. When a significant *P*-value was obtained using one-way ANOVA, the group means were examined by the Holm–Bonferroni method. Values of *P* < 0.05 were considered statistically significant. For the comparison of categorical variables between the groups were used the χ^2^ test.

## 3. Results

### 3.1. Baseline characteristics

The ratio of non-altered myocardial deformation was assessed in 71 patients. The baseline characteristics of these patients have been previously reported, in brief the details are presented in Tables 1, 2 (10, 17–20). The difference in the sex distribution between the groups is not significant (χ^2^ = 5.21, *p* = 0.157, *n* = 71) in Table 1. Due to the non-major numbers in each group, we refrain from further testing. Table 2 shows the increasing global strain values, including both global circumferential (GCS) and longitudinal strain (GLS), with smaller MyoHealth values. Simultaneously, the cardiac index remains on the same level across all groups.

**TABLE 1:** Baseline characteristics by study subgroup.

	Control (*n* = 19) Mean ± SD [median]	HFpEF (*n* = 19) Mean ± SD [median]	HFmrEF (*n* = 19) Mean ± SD [median]	HFrEF (*n* = 18) Mean ± SD [median]	*P*-value
Age–years	59.0 ± 6.84	77.6 ± 8.1	67.0 ± 9.6	64.2 ± 10.1	<0.01
Female sex–no. (%)	9 (47.4)	9 (47.4)	6 (31.6)	3 (16.7)	0.16
LVEF, mean–%	61.6 ± 5.37	61.5 ± 5.87	44.8 ± 2.90	32.9 ± 4.71	<0.01
NT-proBNP–ng/l	88.7 ± 61.1 [79]	586.4 ± 612.1 [314]	790.2 ± 1,138.1 [379]	2,247.5 ± 3,447.3 [886]	<0.01
Presence of CAD–no. (%)	0 (0)	12 (66.7)	15 (78.9)	13 (76.5)	<0.01

CAD, coronary artery disease; HFmrEF, heart failure mid-range preserved ejection fraction; HFpEF, heart failure with preserved ejection fraction; HFrEF, heart failure with reduced ejection fraction; LVEF, left ventricular ejection fraction; n, number; NT-proBNP, N-terminal pro-B-type natriuretic peptide; SD, standard deviation.

**TABLE 2:** Baseline characteristics by the ratio of non-altered LV segments (MyoHealth).

	MyoHealth ≥ 80% (*n* = 7) Mean ± SD [median]	MyoHealth 60–<80% (*n* = 30) Mean ± SD [median]	MyoHealth 40–<60% (*n* = 17) Mean ± SD [median]	MyoHealth < 40% (*n* = 21) Mean ± SD [median]
Age–years	62.9 ± 15.2	71.5 ± 9.1	65.6 ± 11.1	65.6 ± 9.9
Female sex–no. (%)	3 (42.9)	18 (60)	3 (17.6)	3 (14.3)
LVEF, mean–%	62.1 ± 4.1	60.0 ± 8.5	47.1 ± 7.3	36.0 ± 7.3
NT-proBNP–ng/l	387.9 ± 791.3 [63]	341.7 ± 415.6 [254]	498.4 ± 389.9 [366]	2,189.1 ± 3,306.4 [653]
Presence of CAD–no. (%)	1 (14.3)	13 (43.3)	12 (70.6)	14 (66.7)
Cardiac index–l/min/m^2^	2.5 ± 0.3	2.7 ± 1.1	2.7 ± 0.7	2.7 ± 0.5
GCS–%	−19.7 ± 2.3	−17.4 ± 1.6	−14.4 ± 2.6	−10.3 ± 2.1
GLS–%	−20.6 ± 1.6	−19.2 ± 1.1	−16.0 ± 2.2	−11.0 ± 3.4
Controls–no. (%)	5 (71.4)	12 (40.0)	2 (11.8)	0 (0)
HFpEF–no. (%)	2 (28.6)	14 (46.7)	3 (17.6)	0 (0)
HFmrEF–no. (%)	0 (0)	3 (10)	9 (52.9)	7 (33.3)
HFrEF–no. (%)	0 (0)	1 (3.3)	3 (17.6)	14 (66.7)

CAD, coronary artery disease; GCS, LV global circumferential strain; GLS, LV global longitudinal strain; HFmrEF, heart failure mid-range preserved ejection fraction; HFpEF, heart failure with preserved ejection fraction; HFrEF, heart failure with reduced ejection fraction; LVEF, left ventricular ejection fraction; n, number; NT-proBNP, N-terminal pro-B-type natriuretic peptide; SD, standard deviation.

### 3.2. Functional capacity—6MWD

**FIGURE 1:**
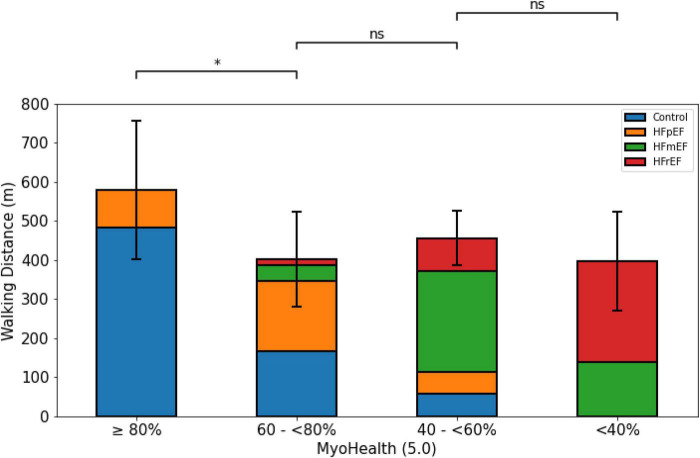
6-minute walk distance across MyoHealth groups. MyoHealth: ratio of myocardial segments with preserved deformation to the total number of myocardial segments. MyoHealth ≥ 80% vs. MyoHealth 60–<80%: *p* = 0.03; MyoHealth 60–<80% vs. MyoHealth 40–<60%: *p* = 0.31; MyoHealth 40–<60% vs. MyoHealth < 40%: *p* = 0.59. *Significant; ns, not significant.

The 6MWD was significantly different across groups separated by the MyoHealth score (overall *p*-value: 0.03). Figure 1 shows the longest 6MWD in the group with the preserved MyoHealth score (MyoHealth ≥ 80%: 579.8 ± 177.6 m) and shorter distances in the other groups (MyoHealth 60–<80%: 401.3 ± 121.7 m; MyoHealth 40–<60%: 456.4 ± 68.9 m; MyoHealth < 40%: 397.6 ± 125.9 m). S2 of Supplementary material highlights the major role of the MyoHealth score in the prediction of the 6MWD when compared to LVEF and the LV global longitudinal strain.

### 3.3. Perceived exertion—Borg scale

**FIGURE 2:**
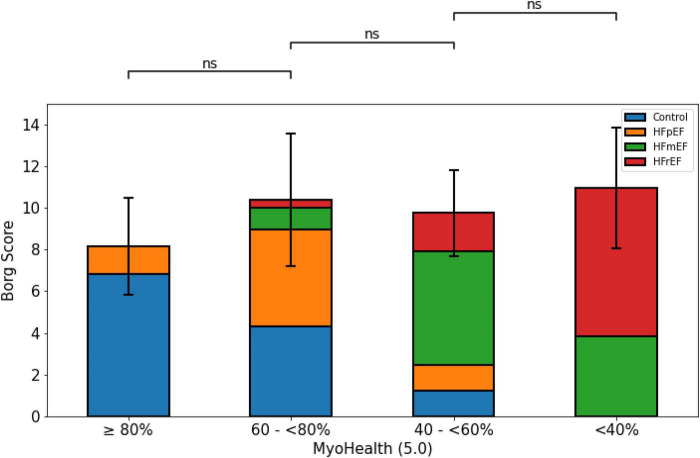
Perceived exertion at the end of the 6-minute walk test across MyoHealth groups. MyoHealth ≥ 80% vs. MyoHealth 60–<80%: *p* = 0.45; MyoHealth 60–<80% vs. MyoHealth 40–<60%: *p* = 1; MyoHealth 40–<60% vs. MyoHealth < 40%: *p* = 0.43. ns, not significant.

Figure 2 illustrates the level of perceived exertion at the end of the 6MWT. The overall comparison revealed no difference between the groups (MyoHealth ≥ 80%: 8.2 ± 2.3 m; MyoHealth 60–<80%: 10.4 ± 3.2; MyoHealth 40–<60%: 9.8 ± 2.1; MyoHealth < 40%: 11.0 ± 2.9; overall *p*-value: 0.20).

### 3.4. Quality of life–MLHFQ

**FIGURE 3:**
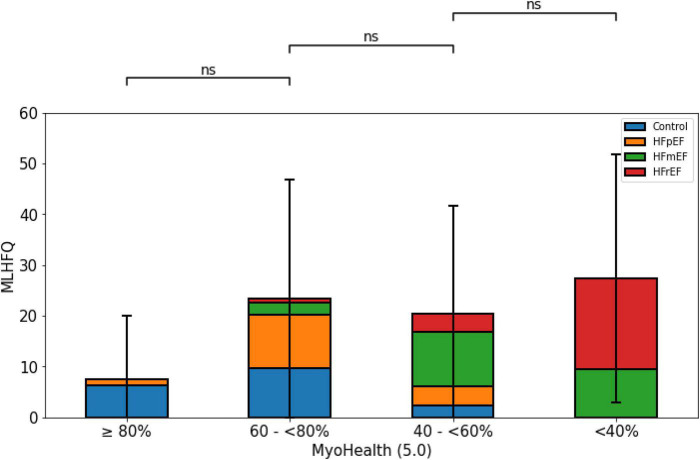
Quality of life by MLHFQ across MyoHealth groups. MyoHealth ≥ 80% vs. MyoHealth 60–<80%: *p* = 0.15; MyoHealth 60–<80% vs. MyoHealth 40–<60%: *p* = 1; MyoHealth 40–<60% vs. MyoHealth < 40%: *p* = 1. ns, not significant.

Figure 3 demonstrates the QoL measure assessed by the MLHFQ with lower numbers indicating a higher QoL. It shows the lowest MLHFQ score values in the group with preserved myocardial deformation. However, the comparison did not reveal a reliable difference across the study population (MyoHealth ≥ 80%: 7.5 ± 12.4; MyoHealth 60–<80%: 23.4 ± 23.4; MyoHealth 40–<60%: 20.5 ± 21.2; MyoHealth < 40%: 27.4 ± 24.4; overall *p*-value: 0.15).

### 3.5. Symptom burden—NYHA functional class

**FIGURE 4:**
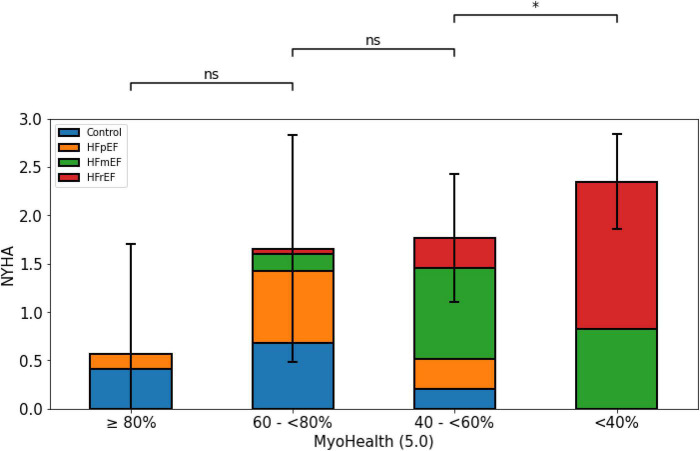
Limitations and symptom burden by NYHA functional class across MyoHealth groups. MyoHealth ≥ 80% vs. MyoHealth 60–<80%: *p* = 0.15; MyoHealth 60–<80% vs. MyoHealth 40–<60%: *p* = 1; MyoHealth 40–<60% vs. MyoHealth < 40%: *p* < 0.01. *Significant; ns, not significant.

Figure 4 displays the significant association of the NYHA functional class and the proportion of preserved myocardial segments. While the NYHA class was lowest in the group with a preserved MyoHealth score, it was similarly higher in the groups with decreased score values (MyoHealth ≥ 80%: 0.6 ± 1.1; MyoHealth 60–<80%: 1.7 ± 1.2; MyoHealth 40–<60%: 1.8 ± 0.7; MyoHealth < 40%: 2.4 ± 0.5; overall *p*-value < 0.01).

## 4. Discussion

In this study, aiming to better characterize HF patients and imaging parameters indicating their symptom burden, we found that the proportion of myocardial segments with preserved myocardial deformation indicates better functional capacity. Furthermore, this exploratory analysis suggests an association of the MyoHealth score with quality of life surrogate parameters in larger study population.

Table 2 shows the consistency of our data. The cardiac index remains on a similar level across all groups who were either healthy subjects or HF patients in a stable outpatient condition. The global strain values (GCS and GLS) were increasing with smaller MyoHealth score values, as the MyoHealth score *per se* represents the proportion of segments with preserved strain values.

Our observational study of consecutive patients with HF as well as control subjects was carefully designed to better characterize myocardial contraction. After focusing on the contraction pattern and describing the onset of changes in HF in interventricular septal segments, we sought to highlight that CMR scans in HF are not only relevant to characterize HF but to better phenotype patients with regards to parameters limiting their lives on a daily basis (10).

Given the high prevalence of HF and a nearly half of these patients showing nearly normal values with regards to traditional HF parameters, e.g., LVEF, there is an unmet need for innovative tools to diagnose patients and identify those at risk (8). In this analysis we sought to identify a parameter that reflects both an insight to the cardiac contraction with its regional differences and key parameters limiting patients’ everyday experience, functional capacity, and quality of life (8, 25).

The MyoHealth score allows for the estimation of the clinical condition when functional capacity data is not available. If the signal with regards to the QoL assessed by the MLHFQ prove feasible and the differences are significantly different in a larger study population, this might lead to fewer resources than assessing the symptom burden systematically aside from the image acquisition, e.g., by conducting a 6MWT or questionnaires for QoL.

Impaired functional capacity, often assessed by 6MWT, is the main symptom in patients with HF, regardless of LVEF (26–28). With the 6MWD being relevantly reduced in both patients and subjects with a reduced ratio of segments with preserved myocardial deformation, as seen in Figure 1, we could show the association of an innovative parameter reflecting the myocardial contraction with its regional disparities. The validity of this finding is shown in Figure 2, as patients in all groups reached a comparable level of exhaustion indicated by values on the Borg scale. The comparable values on the Borg scale indicate that patients were tested with the same rigor to maintain a comparable, adjusted intensity to test their capacities.

While there is some evidence indicating a worse prognosis based on CMR characteristics in HFpEF, the association with the symptom burden remains to be further explored (29–31).

The HFA-PEFF algorithm introduced imaging parameters to diagnose HFpEF, e.g., E/e’, tricuspid regurgitation velocity, left atrial volume index, and LV wall thickness (32). In brief, the HFA-PEFF algorithm leads to a score value for an individual patient based on functional, morphological and biomarker parameters, that may diagnose HFpEF, exclude HFpEF or indicate further investigation for a diagnosis by stress testing. Many of the highlighted functional and morphological parameters are derived from echocardiography and cannot be acquired reliably by routine CMR techniques. Nonetheless, it has been shown that the application of a CMR stress testing technique is a feasible strategy for further investigations in suspected HFpEF patients (33). All these efforts aim to better understand patients with a symptom burden leading to a suspected diagnosis of HF and a preserved traditional parameter, LVEF. Identifying those with altered cardiac function, like our analysis of regional differences of myocardial deformation, is at the core of recent findings, which aspire to lead to an earlier diagnosis as well as a better differentiation of the broad spectrum of patients diagnosed with HFpEF.

HFA-PEFF parameters as well as other HFpEF criteria have been often analyzed for a prognostic value regarding survival, but only recently a few studies focused on functional capacity (26, 34, 35). These analyses focused on clinical features and their impact on the 6MWT (26). Clinical information is often neither at the disposal of the physician reporting on cardiac imaging nor of the team managing the patient, which stresses the relevance of this analysis highlighting a method to increase the robustness of images to interpret.

With regards to the QoL the greatest difference could be observed between the group with preserved MyoHealth score and the impaired groups, as illustrated in S1, indicating that this parameter indicates QoL. While many patients with HF suffer from depression, QoL was only recently used as a clinical trial outcome parameter in HF—very rarely in cardiac imaging and CMR studies (36–40).

Figure 4 reflects the harmony of the presented data, as the subjects with a preserved ratio of altered myocardial deformation are those with the lowest symptom burden with regards to the subjective NYHA classification.

### 4.1. Limitations

The main limitation of our study is that we cannot provide prognostic information of the subjects and patients examined. Nonetheless, the main objective of this analysis was to evaluate the predictive value of regional myocardial deformation on functional capacity as well as the quality of life in HF patients, especially HFpEF.

However, our explorative analyses to better understand the differences between the study subgroups are limited due to the smaller subgroup sizes the more fragmented they become. Comparing HF entities from different subgroups against each other within our analyses (Figures 1–4) will include subgroups with very few subjects, which restricts massively the claim of transferability. Further analyses of our exploratory indications require replication in larger cohorts. Due to this restriction, we also did not perform a *post hoc* subject sex or age matched analysis, as it would lead to even smaller numbers of subgroup subjects.

The small numbers also limit some of our analyses where we see quantitative difference, which do not reach the level of statistical significance, e.g., the assessment of QoL by the MLHFQ. Revisiting this analysis in a larger study population would lead to results that are more reliable. However, we believe that these not significant results are a signal to further analyses.

## 5. Conclusion

The results of this study promise an association between regional LV strain impairment and the symptom burden of HF patients with regards to functional capacity and quality of life, especially relevant in patients with a preserved LVEF that requires future prospective validation.

## Data availability statement

The original contributions presented in this study are included in this article/Supplementary material, further inquiries can be directed to the corresponding author.

## Ethics statement

The studies involving human participants were reviewed and approved by Ethikkommission der Charité – Universitätsmedizin Berlin. The patients/participants provided their written informed consent to participate in this study.

## Author contributions

DH, HDD, FE, and SK: conception and design of the study and literature review. DH, MB, and SK: analysis and interpretation of the data. DH: drafting of the manuscript. DH, PD, and MB: data collection. All authors revising and editing the manuscript and approved the submitted version.
