# Supplementary material for: Reduced functional capacity is associated with the proportion of impaired myocardial deformation assessed in heart failure patients by CMR
Source: Front Cardiovasc Med. 2023 Feb 9;10:1038337. doi: 10.3389/fcvm.2023.1038337 (PMC9947709; doi:10.3389/fcvm.2023.1038337)
Supplement: Supplementary file 1 [file Data_Sheet_1.docx]

**Supplementary material**

S1 – MyoHealth score illustrated


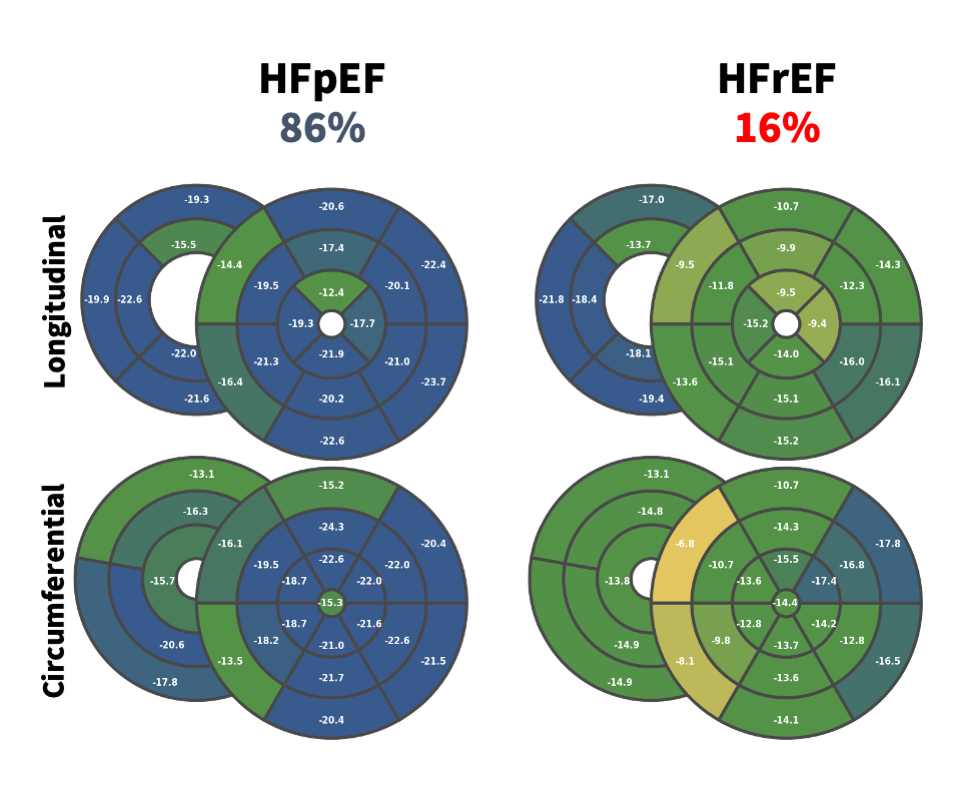


HFpEF: heart failure with preserved ejection fraction; HFrEF: heart failure with reduced ejection fraction.

These figures illustrate the regional myocardial strain segments and their level of alteration from the reference values in a color-coded system. Blue indicates normal values of myocardial deformation, green reduced strain values (strain > - 17 %) and yellow more reduced strain values in the respective segment.^1,2^

There are 37 segments of the left ventricle (LV) (16 segments with strain assessement in the longitudinal axis and 21 segments in the circumferential axis) and 11 segments for the right ventricle (6 segments in the longitudinal and 5 segments in der circumferential axis).^1,2^

In this figure, there are two cases presented:

- HFpEF: 5 out of the 37 LV segments show relevantly reduced myocardial deformation indicated by an altered regional strain value. Therefore 28 out of the 33 segments are within the reference range. MyoHealth score: 32/37 = 86%.
- HFrEF: 31 out of the 37 LV segments show altered strain values, 6 segments are within the reference range. MyoHealth score: 6/37 = 16%.

S2 – Multiple regression comparing MyoHealth score, LV strain and LVEF

| **Coefficients^a^** | | | | | | |
| --- | --- | --- | --- | --- | --- | --- |
| Model | | Unstandardized Coefficients | | Standardized Coefficients | t | Sig. |
|  |  | B | Std. Error | Beta |  |  |
| 1 | (Constant) | 465.952 | 115.350 |  | 4.039 | <.001 |
|  | MyoHealth | 2.216 | 1.698 | .419 | 1.305 | .196 |
|  | LV.GLS | .929 | 11.017 | .026 | .084 | .933 |
|  | LVEF | -2.970 | 2.093 | -.302 | -1.419 | .161 |
| a. Dependent Variable: WalkDistance | | | | | | |

LVEF: left ventricular ejection fraction; LV.GLS: left ventricular global longitudinal strain; Sig: significance level, p-value; Std. Error: standard error.

In this multiple regression model including the MyoHealth score, LV strain values (LV global longitudinal strain, LV.GLS) and LVEF, assessing the standardized coefficients highlights the relatively important value of the MyoHealth score in predicting the 6MWD as leading among these three variables (Beta = 0.419) followed by the LVEF (Beta = -0.302) and the LV.GLS (Beta = 0.026). However, with the limitations of this analysis and the sample size we appreciate the order between these three parameters but consider the values *per se* as secondary relevant information.

1. Hashemi D, Motzkus L, Blum M, Kraft R, Tanacli R, Tahirovic E, Doeblin P, Zieschang V, Zamani SM, Kelm M, et al. Myocardial deformation assessed among heart failure entities by cardiovascular magnetic resonance imaging. *ESC Heart Failure*. 2021. doi: 10.1002/ehf2.13193

2. Steen H, Montenbruck M, Gerzak B, Schwarz AK, Kelle S, Giusca S, Korosoglou G, Dent S, Lenihan D. Cardiotoxicity During Cancer Treatment Causes More Regional Than Global Dysfunction: the Prefect Study. *Journal of the American College of Cardiology*. 2020;75:1824-1824. doi: 10.1016/s0735-1097(20)32451-7
